# Endothelin-1-mediated miR-let-7g-5p triggers interlukin-6 and TNF-α to cause myopathy and chronic adipose inflammation in elderly patients with diabetes mellitus

**DOI:** 10.18632/aging.204034

**Published:** 2022-04-25

**Authors:** Chung-Huang Tsai, Pei-Ju Huang, IT Lee, Chien-Min Chen, Min Huan Wu

**Affiliations:** 1 Department of Family Medicine, Chung-Kang Branch, Cheng Ching Hospital, Taichung, Taiwan; 2 Center for General Education, Tunghai University, Taiwan; 3 Bachelor of Science in Senior Wellness and Sport Science, Tunghai University, Taiwan; 4 Department of Family Medicine, Changhua Christian Hospital, Changhua, Taiwan; 5 Division of Endocrinology and Metabolism, Department of Internal Medicine, Taichung Veterans General Hospital, Taichung, Taiwan; 6 School of Medicine, National Yang Ming Chiao Tung University, Taipei, Taiwan; 7 School of Medicine, Chung Shan Medical University, Taichung, Taiwan; 8 Division of Traditional Chinese Medical, Sinying Hospital, Tainan, Taiwan; 9 Senior Life and Innovation Technology Center, Tunghai University, Taiwan; 10 Life Science Research Center, Tunghai University, Taiwan

**Keywords:** diabetes, sarcopenia, miRNA, endothelin-1 (ET-1), TNF-α, interleukin-6, hyperglycemia

## Abstract

Background: Diabetes and sarcopenia are verified as mutual relationships, which seriously affect the quality of life of the elderly. Endothelin-1 is well investigated, is elevated in patients with diabetes, and is related to muscle cellular senescence and fibrosis. However, the mechanism of ET-1 between diabetes and myopathy is still unclear. The aim of this study was to evaluate the prevalence of sarcopenia in the elderly with diabetes and to clarify its relationship with ET-1 molecular biological mechanism, progress as well as changes in muscle and fat.

Methods: We recruited 157 type 2 diabetes patients over 55 years old and investigated the prevalence of sarcopenia in diabetes patients and examined the association of ET-1 alterations with HbA1c, creatinine, or AMS/ht2. Next, sought to determine how ET-1 regulates inflammation in muscle cells by western blot and qPCR assay. Using XF Seahorse Technology, we directly quantified mitochondrial bioenergetics in 3T3-L1 cells.

Results: ET-1 was positively correlated with HbA1c, creatinine levels, and duration of disease, and negatively correlated with AMS/ht2. We found that ET-1 dose-dependently induces tumor necrosis factor-α (TNF-α) and interleukin (IL)-6β expression through the PI3K/AKT, and NF-κB signaling pathways in C2C12 cells. Also identified that TNF-α, IL-6β, and visfatin releases were found in co-cultured with conditioned medium of ET-1/C2C12 in 3T3-L1 cells. ET-1 also reduces the energy metabolism of fat and induces micro-environment inflammation which causes myopathy. ET-1 also suppresses miR-let-7g-5p expression in myocytes and adipocytes.

Conclusion: We describe a new mechanism of ET-1 triggering chronic inflammation in patients with hyperglycemia.

## INTRODUCTION

In an aging society, the prevalence of diabetes and musculoskeletal diseases is increasing annually [1, 2]. Body composition changes during aging, resulting in significant losses of skeletal muscle mass and increased body fat percentage [3]. Collectively and independently, low muscle mass and adiposity are associated with higher incidence of metabolic disorders, including T2DM [4]. “Sarcopenia” describes age-related declines in muscle mass and function and has been implicated as both a cause and consequence of T2DM [5]. When sarcopenia and obesity coexist, this condition is called sarcopenic obesity (SO). SO is a geriatric syndrome and a critical risk factor for death among elderly [6, 7]. T2DM is characterized by insulin resistance, increased advanced glycation end-products (AGEs), a proinflammatory phenotype and oxidative stress, which can lead to micro- and macro-vascular complications [8, 9].

The prevalence of sarcopenia in T2MD is reported to be high, especially in patients with poor glycemic control [4–6]. Skeletal muscle inflammation is the key to muscle loss and an important step in sarcopenia. Generally, as age and function declines, lean body mass will decrease and on the contrary body fat will increase. Common indicators of sarcopenia include appendix skeletal muscle mass (ASM), total skeletal muscle mass (TSMM), lean body mass (LBM), and fat-free mass (FFM). As reference methods are not available for identifying low skeletal muscle mass in clinical practice, the European Group on Sarcopenia in Older People (EGSOP) the Asian Working Group for Sarcopenia (AWGS) and the International Consensus for Cancer Cachexia (ICCC) guidelines accept bioelectrical impedance analysis (BIA) as an option for sarcopenia and cachexia assessment [10]. According to the study of AWGS 2019 Consensus Update on Sarcopenia Diagnosis and Treatment, bioelectrical impedance analysis (BIA) and ASM divided by height squared (ASM/ht^2^) are the most commonly used measurement methods for sarcopenia [11]. AWGS 2019 retains the original cutoffs for height-adjusted muscle mass: bioimpedance, <7.0 kg/m^2^ in men and <5.7 kg/m^2^ in women.

Glycated hemoglobin (HbA1c) is a critical index of T2DM and is an invaluable item in general health examination or hospital routine. Its high value is associated with chronic diseases such as diabetes and cardiovascular disease. Previous research shows that there is a U-shaped relationship between the concentration of HbA1c and the decline of physiological function in advanced age [12], especially for a high concentration of soluble interleukin 6 (IL-6) in the blood [13]. The rate of decline in body function, essential to assessing aging, is also related to the concentration of HbA1c and related [14–16]. Previously report also indicated that plasma endothelin-1(ET-1) levels increased in T2MD and predicts incident diabetic peripheral neuropathy [17]. ET-1 is an effective vasoconstrictor peptide. The plasma ET-1 concentration level in diabetic patients is positively correlated with microangiopathy. ET-1 is also significantly increased in obesity [18, 19]. Chronic inflammation causes obesity through several pathways, including releasing inflammatory cytokines to recruit adipose tissue macrophages (ATMs), stimulating lipogenesis, and changing metabolic capacity [20, 21]. Plasma creatinine is often used as a parameter of muscle damage in clinical practice. In recent years, many studies have pointed out that it can also be used as an indicator of sarcopenia and related diseases such as diabetes [22, 23].

MicroRNAs (miRNAs) have attracted attention as potential biomarkers and targets for specific therapies. MiRNAs are small non-coding RNAs (21–25 bases) that are not translated into proteins but inhibit the function of their target messenger RNAs (mRNAs) by destabilizing them and inhibiting their translation. Previous studies have shown that miRNAs play pivotal roles in the development of sarcopenia. Many miRNAs increase or decrease in muscles and blood of patients with sarcopenia and rodent models of sarcopenia. Additionally, several miRNAs have been demonstrated to affect sarcopenia in myocytes *in vitro* or rodent sarcopenia models *in vivo* [24]. A large number of studies reporting the dysregulation of miRNAs in the serum/plasma of patients with diabetes have emerged in the past decade. In a global profile study focusing on T2D circulating miRNAs, approximately 70 miRNAs showed elevated levels, and about 100 miRNAs showed reduced levels in blood samples of T2D patients [25]. A meta-analysis confirmed 40 significantly dysregulated miRNAs in T2D patients [26]. These results suggest that miRNAs are potential biomarkers and targets of gene therapy for sarcopenia and DM.

There are many publications describing the involvement of low-grade inflammation in the aging process and age-related diseases [27, 28]. To explain its diverse implications, various terms and views have been proposed. Included are inflammaging, molecular inflammation, micro-inflammation, pan-inflammation, and gero-inflammation, all of which describe the increased chronic inflammatory activity and proinflammatory mediators associated with aging, but at present, these age-related chronic inflammation phenomena still remain poorly defined and uncharacterized [29]. All metabolic diseases may originate from chronic inflammation, such as T2MD, obesity and sarcopenia, usually occur simultaneously, however, the mechanism between T2MD and OS is still unclear. Therefore, the aim of this study is to investigate the relationship between HbA1c, ET-1, Creatinine, and muscle mass in T2MD elderly patients; to further understand the underlying molecular mechanism between myocyte and adipocyte, which will provide a supplementary reference for clinical diagnoses in the future.

## MATERIALS AND METHODS

### Materials

Cell culture supplements were purchased from Invitrogen (Carlsbad, CA, USA). Antibodies against IL-6 (SC-28343), p-p65 (Ser536; SC-101752), p-65 (SC-8008), p-85 (SC-1637), p-AKT (Thr308; SC-16646-R), AKT (SC-5298), p-IKKα/β (Ser180/Ser181; SC-23470-R), IKKα/β(SC-7607), p-IκBα (Ser32; SC-8404), IκBα (SC-203), PCNA(SC-36), and β-actin (SC-58673), and C6 ceramide (an ERK activator), were all purchased from Santa Cruz (Santa Cruz, CA, USA). Anti-TNF-α (a11534) was obtained from Abclonal (Woburn, MA, USA). Antibody against p-p85 (Tyr458/Tyr199; 4228S) was purchased from Cell Signaling (Danvers, MA, USA). AKT inhibitor were supplied by Calbiochem (San Diego, CA, USA). Ly294002, TPCK and PDTC were bought from Enzo Life Sciences International (Plymouth Meeting, PA, USA) and Cell culture supplements were purchased from Invitrogen (Carlsbad, CA, USA). Dual-Luciferase^®^ Reporter Assay System was bought from Promega (Madison, WI, USA). All other chemicals not described above were supplied by Sigma-Aldrich (St Louis, MO, USA).

### Cell culture

The murine myoblast cell lines C2C12, G7, and 3T3-L1 were purchased from American Type Culture Collection (Manassas, VA, USA). C2C12 and G7 cells were cultured in Dulbecco’s Modified Eagle Medium (DMEM) supplemented with 10% fetal bovine serum, penicillin (50 units/mL), and streptomycin (50 μg/mL), as previously described [30–32]. ET-1–conditioned media (CM) were prepared from the supernatant of C2C12 or G7 treated with ET-1. 3T3-L1 preadipocytes (CL-173™; ATCC) were cultured in Dulbecco’s modified Eagle’s medium (DMEM) (Invitrogen, Carlsbad, CA, USA) containing 10% fetal bovine serum (FBS) and 10 mg/ml penicillin/streptomycin in an atmosphere of 10% CO_2_ at 37°C. 3T3-L1 cells were then co-cultured with the CM for 24 h. Next, the cellular lipid content was assessed by Oil Red O staining (Sigma-Aldrich).

### Patient consent and clinical samples

Blood samples were obtained from patients aged over 55 years with diabetes mellitus at T2DM and were defined as having fasting capillary blood glucose level (>6.1 mmol/L) or plasma glucose measurement (FPG > 7.0 mol/L) in accordance with World Health Organization (WHO) 2006 criteria. Exclusion criteria included who had a history of cerebral stroke, heart stents, artificial pacemakers or other metal implants implanted in the body, or had malignant tumors, hepatopathy, end-stage renal disease, thyroid gland dysfunction, arthrophlogosis, carpal tunnel syndrome, or had taken special nutritional supplements such as protein powder in recent three months. HbA1c was measured by high-performance liquid chromatography using DCCT-aligned methods. For each patient, intrapersonal mean and SDs of all recorded HbA1c measurements were calculated. This sample comes from Taichung Veterans General Hospital, Cheng Ching Hospital, Singin Hospital, and Changhua Christian Hospital, Taiwan. The study protocol was approved by the Institutional Review Board (IRB: CG16183A-2) of Taichung Veterans General Hospital, and all procedures were performed in accordance with the IRB’s guidelines. Informed written consent was obtained from all patients. Clinical parameters and disease characteristics for each subject were collected according to a standardized protocol. Recorded clinical parameters included age, gender, body mass index (BMI), systolic blood pressure (SBP) and diastolic blood pressure (DBP), endothelin-1(ET-1), creatinine, and Hemoglobin A1c (HbA1c). Disease characteristics included self-reported duration of diabetes mellitus and family history.

### RT-qPCR of mRNA and miRNA

Total RNA was extracted from C2C12 and G7 cells using the TRIzol reagent (MDBio, Taipei, Taiwan), according to the manufacturer’s instructions. The RNA concentration was determined using a Nanovue™ spectrophotometer (GE Healthcare). RNA was converted into complementary DNA (cDNA) by M-MLV reverse transcription (Invitrogen, Thermo Fisher Scientific), following the manufacturer’s instructions. The qPCR analysis was performed as per the manufacturer’s protocols. cDNA was amplified with the forward (F) and reverse (R) primers by PCR. IL-6 was 5′-GACAACTTTGGCATTGTGG-3′ (F) and 5′-ATGCAGGGATGATGTTCTG-3′ (R), ET-1 were 5′-GTGGAAGGAAGGAAACTAC-3′ (F) and 5′-CAAGAAGAGGCAGAAAGGCA-3′ (R). TNF-α Sense 5′-CCGATGGGTTGTACCTTGTC-3′ (F) and 5′-CAAGAAGAGGCAGAAAGGCA-3′ (R). [33–35]. miRNA mmu-let-7g-5p UGAGGUAGUAGUUUGUACAGUU.

### Western blot analysis

The cell lysate was separated by SDS-PAGE electrophoresis, and proteins were then transferred to polyvinylidene difluoride membranes, following the method described in our previous work [36, 37]. After blocking, the membranes were incubated with primary antibody and then secondary antibody. For enhanced chemiluminescent imaging, the blots were visualized with the UVP Biospectrum system (UVP, Upland, CA, USA) [38–40].

### ELISA assay

C2C12 and 3T3-L1 cells were cultured and stimulated with ET-1 for 24 h with or without the transfection of siRNAs or inhibitors. Serum and the CM were collected, and ET-1 levels were quantified using an ET-1 ELISA kit (Peprotech, Rocky Hill, NJ, USA).

### XF24 oxygen consumption analysis

3T3-L1 fibroblasts were seeded in Seahorse (Seahorse Bioscience Inc., Billerica, MA, USA) plates at a density of 50,000 cells per well before being differentiated to adipocytes as outlined above. Prior to all assays, cell media were changed to unbuffered DMEM (DMEM base medium supplemented with 25 mM glucose, 1 mM sodium pyruvate, and 1 mM GlutaMax; pH 7.4) and incubated at 37°C in a non-CO_2_ incubator for 60 min. Cellular bioenergetics, mitochondrial function assays, and parameter calculations were performed as previously described using the Seahorse XF24 analyzer. For assessing oxygen consumption responses to norepinephrine, four basal measurement periods were performed prior to the injection of norepinephrine (1 μM final) or vehicle (H_2_O), and seven subsequent measurements were taken as previously described [41].

### Plasmid construction and luciferase assays

C2C12 and G7 cells were transfected with a reporter plasmid using Lipofectamine 2000 (Invitrogen) according to the manufacturer’s recommendations. At 24 h after transfection, the cells were pretreated with inhibitors for 30 min, and then, ET-1 or vehicle was added for 24 h. Cell extracts were then prepared, and luciferase and β-galactosidase activities were measured. Wild-type (wt) Twist-3'-UTR was constructed into the pGL2-Control vector. The predicted Ikbkb binding site for miRNA was identified by the Targetscan (http://www.targetscan.org/). Mutant plasmids that attenuate the interaction between Ikbkb 3′UTR and miRNA were generated using a QuikChange Site-Directed Mutagenesis kit (Stratagene, Cedar Creek, TX, USA). These plasmids were transfected into cells using Lipofectamine 2000.

### Statistics

All values are given as mean ± standard error of the mean (SEM). The relationship between annual HbA1c values and time was determined by estimating Spearman’s correlation for patients subgrouped by HbA1c level. Statistical analysis was performed using SPSS version 22.0 (IBM Corp., Armonk, NY, USA). Student’s *t*-test was used to assess between-group differences. In order to investigate the differences between HbA1c, BIA levels, a one-way analysis of variance (ANOVA) was applied in these groups. Bonferroni correction was used to account for multiple pairwise comparisons. A *p* value of <0.05 was considered statistically significant.

## RESULTS

### Characteristics of enrolled patients categorized based on Hba1c

**Figure 1:**
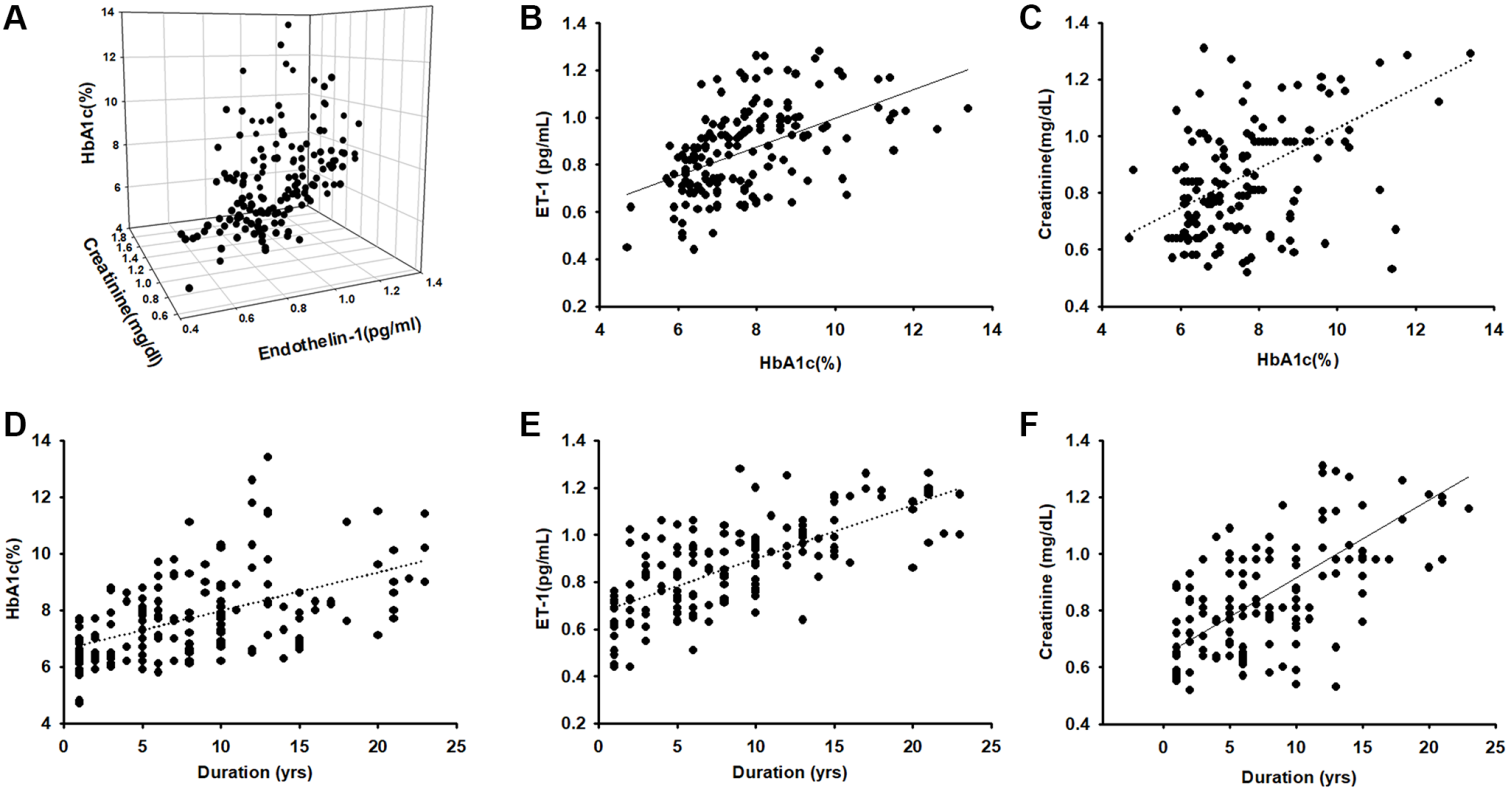
**Endothelin-1(ET-1)/creatinine expression is positively correlated with HbA1c expression in elderly patients with diabetes mellitus (DM).** (**A**–**C**) ELISA analysis indicates higher serum ET-1 and creatinine levels with higher HbA1c(R = 0.74). (**D**) Correlation between levels of HbA1c and diseases duration in elderly patients with DM (R = 0.58). (**E**) Serum ET-1 was higher with longer disease duration and lower bioelectrical impedance analysis values (R = 0.71). (**F**) Correlation between levels of serum creatinine and diseases duration with DM (R = 0.68).

A total of 157 patients aged >55 years with obesity and diabetes diagnosed by a specialist in metabolism or family medicine were enrolled into this study and completed BIA and blood parameter assessments. The mean age of the patients was 66.82 ± 8.83 years; 40.63% were men and 59.38% women; weight was 69 ± 10 kg and height 160 ± 10 cm. The patients were assigned to four groups based on the plasma HbA1c range: HbA1c < 6.5%, HbA1c = 6.5%–7.5%, HbA1c = 7.5%–8.5%, and HbA1c > 8.5%. Table 1 presents the characteristics of the participants, including body composition and serum parameters. Disease duration in years, plasma creatinine, and ET-1 were positively correlated (*p* < 0.05) with HbA1c levels. Disease duration and HbA1c were negative related with ASM/ht^2^ (BIA) and muscle mass. We further to analyzed the Pearson correlation between ET-1 and other parameters and found that ET-1 levels have a positively correlated with diabetes duration, plasma creatinine HbA1c and body fat percentage but negative with ASM/ht^2^. Furthermore, compared with the ET-1, creatinine, and HbA1c there are correlated in different levels. As shown in Figure 1, the duration of disease is clearly an essential issue in metabolic syndrome (Figure 1D–1F), and the hyperglycemia environment is associated with high ET-1 and creatinine levels (Figure 1A–1C).

**Table 1:** Physiological parameters of DM patients by HbA1c group classification.

	**HbA1c (<6.5)**	**HbA1c (6.5–7.5)**	**HbA1c (7.5–8.5)**	**HbA1c (>8.5)**	**F**	***p***
**Anthropometry**	***N* = 40**	***N* = 36**	***N* = 40**	***N* = 41**		
Male	26	28	17	17		
Female	30	35	24	24		
Age (yr)	64.76 ± 11.68	65.35 ± 10.94	63.65 ± 11.05	63.38 ± 9.87	1.178	0.24
Weight (kg)	69.49 ± 12.92	68.6 ± 11.61	66.02 ± 13.44	74.72 ± 18.23	1.335	0.114
Height (cm)	163 ± 8	161 ± 8	159 ± 9	164 ± 7	0.229	1.000
BMI (kg/m^2^)	26.6 ± 4.17	24.14 ± 1.05	25.78 ± 3.74	26.12 ± 5.69	0.320	1.000
Muscle(kg)	26.03 ± 3.87	26.13 ± 3.61	26.35 ± 7.07	22.93 ± 7.26	1.620	0.019
Fat%	30.69 ± 6.99	30.89 ± 6.6	33.13 ± 5.6	32.48 ± 6.3	1.419	0.067
ASM/ht^2^	10.64 ± 1.64	9.39 ± 1.54	9.31 ± 1.47	8.51 ± 1.00	1.76	0.007
Duration of disease (y)	4.8 ± 3.73	7.59 ± 5.59	8.58 ± 4.22	9.13 ± 5.6	2.059	0.001
Creatinine (mg/dl)	0.75 ± 0.24	0.83 ± 0.19	0.83 ± 0.22	0.96 ± 0.38	1.932	0.002
Endothelin-1 (pg/ml)	0.69 ± 0.12	0.81 ± 0.12	0.83 ± 0.15	0.91 ± 0.12	2.352	0.000

Demographic and clinical characteristics by HbA1c concentration. The mean age of the patients was 66.82 ± 8.83 years; 40.63% were men. The mean course of diabetes was 7.35 ± 5.95 years. Results of the univariate analysis indicated muscle (kg), fat (%), BIA, duration of disease (y), creatinine (mg/dL), and endothelin-1 (pg/mL) were significantly correlated with HbA1c. ^*^p < 0.001.

### ET-1 expression is positively correlated with age, HbA1c, creatinine, and myopathy in diabetes

**Figure 2:**
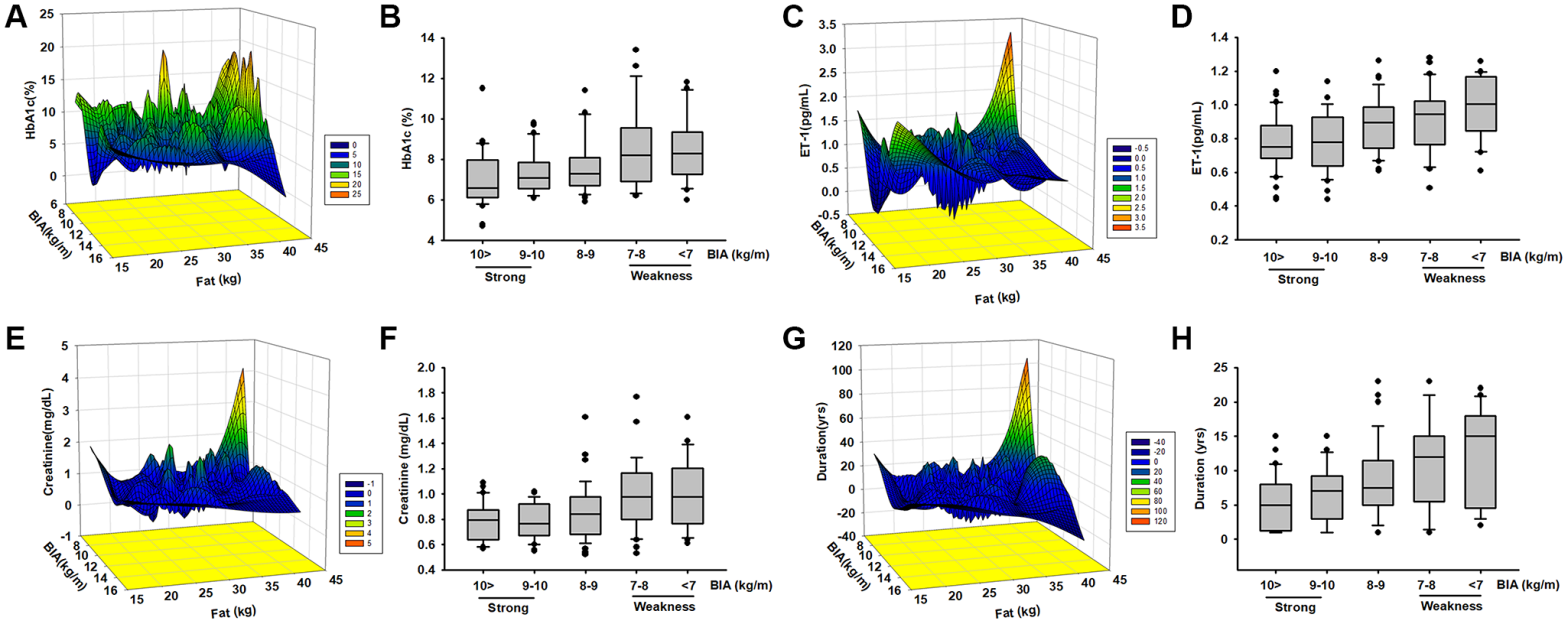
**The levels of HbA1c, ET-1, creatinine expression, and diseases duration are correlated with ASM among elderly patients with diabetes mellitus (DM).** Analysis the correlated the levels of HbA1c (**A**, **B**), ET-1 (**C**, **D**), creatinine (**E**, **F**), and diseases duration (**G**, **H**) with different body composition (ASM/ht2) by mesh graphs and different muscle mass in elderly DM patients.

In order to clarify the relationship between muscle mass and biomarkers we further regroup different muscle mass levels and compare all the parameters. According to EWGSOP and AWGS that muscle mass loss is a threat to elders, the subjects were randomly divided into 5 groups according to ASM/ht^2^ levels (10>, 10–9, 9–8, 8–7, 7<, which of appendicular skeletal muscle mass/ squared height). We analyzed the correlations with serum concentration of ET-1, HbA1c, creatinine, and disease duration. A positive correlation was found between the four main factors of disease duration, and ET-1, HbA1c, and creatinine concentration (R = 0.74). Next, we examined the relationship between HbA1c, ET-1, Creatinine, and ASM/ht^2^. Here, we followed the recommendations of IWGS and used ASM/ht^2^. ASM/ht^2^ are also associated with HbA1c (Figure 2B), ET-1 (Figure 2D), Creatinine (Figure 2F) levels, and disease duration in Figure 2H.

In order to further explore the possible association between HbA1c, ET-1, Creatinine, and the duration of disease were correlated with body composition we used a mesh graph to directly observe these parameters. We noticed that the key point is that the duration of more than 15 years; here, the content of adipose tissue and HbA1c is much higher than those in other subjects (Figure 2A). Comparison of the relationship between ET-1, Creatinine, Duration, and myopathy index creatinine revealed a positive correlation between them (Figure 2C, 2E and 2G). These results indicate that the metabolic factors might be triggered in an individual’s microenvironment. Over time, ET-1 and related factors become unbalanced. ET-1 especially affects the elderly population because it accumulates over time. These data provide strong evidence that ET-1 is a key factor in various conditions and may trigger abnormal physiology and pathophysiology. However, additional experimentation is required to define the connection. Next, we explored the role of ET-1 in muscle cells and adipocytes *in vitro*.

### ET-1 Increases IL-6 and TNF-α expression in myoblast cell lines C2C12 and G7

**Figure 3:**
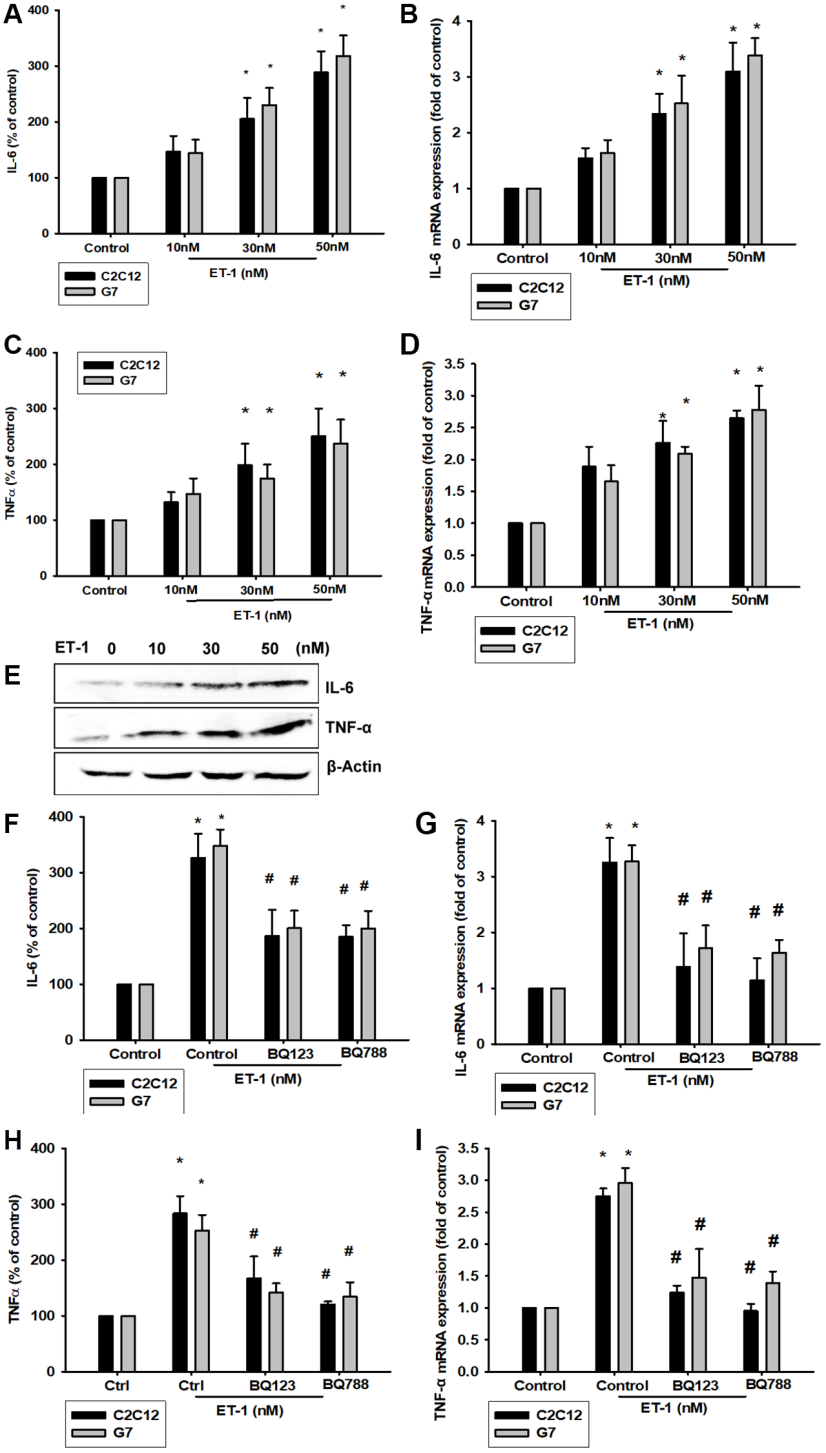
**ET-1 induces IL-6 and TNF-α mRNA and protein levels through ET receptor pathways in myocyte cells (C2C12 and G7 cell lines).** (**A**–**E**) Cells were incubated with ET-1 (10–50 nM) and the levels of IL-6 and TNF-α mRNA and protein expression were examined by EILSA, qPCR, and Western blot assays. (**F**–**I**) Cells were pretreated with ETRs inhibitors BQ123 (5 μM) and BQ788 (5 μM) then stimulated with ET-1. The IL-6 and TNF-α mRNA and protein expression levels were examined by EILSA and qPCR assays. Results are expressed of four independent experiments performed in triplicate. ^*^p < 0.05 compared with control; ^#^*p* < 0.05 compared with the ET-1–treated group.

Previous studies have revealed that ET-1 in the microenvironment leads to several types of inflammation-related conditions and diseases [42, 43]. Therefore, ET-1 may be involved in regulating the muscle inflammation response mechanism. We hypothesized IL-6 and TNF-α may be involved in ET-1–induced inflammatory myopathy. Treatment of myoblast cell lines (C2C12 and G7) with ET-1 increased transcriptional expression of IL-6 and TNF-α, as measured by qPCR (Figure 3B and 3D). ET-1 increased protein expression of IL-6 and TNF-α in C2C12 and G7 cells in a dose-dependent manner in Western blotting (Figure 3E). These cytokine levels in the supernatant from CM of ET-1 were also determined by an ELISA assay (Figure 3A and 3C). Data indicated that ET-1 increased IL-6 and TNF-α mRNA and protein levels. Previous studies have determined that ET-1 affects cells migration through the ET_A_R [44–48]. We hypothesize that the ETR-signaling pathway may be involved in ET-1–stimulated inflammatory myopathy. Furthermore, IL-6 and TNF-α transcription activity was prevented by the ET receptor antagonists BQ123 and BQ788; pretreatment of C2C12 and G7 cells for 30 min with the ETR antagonist BQ123 (5 μM) or BQ788 (5 μM) markedly inhibited ET-1–induced IL-6 and TNF-α expression. (Figure 3F and 3H) mRNA levels were measured by qPCR and secreted protein concentration by ELISA assay (Figure 3C–3I). Together, the results confirmed that ET-1 induces IL-6 and TNF-α upregulation in C2C12 and G7 myoblast cells.

### ET-1 promotes IL-6 and TNF-α production through PI3K and Akt signaling pathways

**Figure 4:**
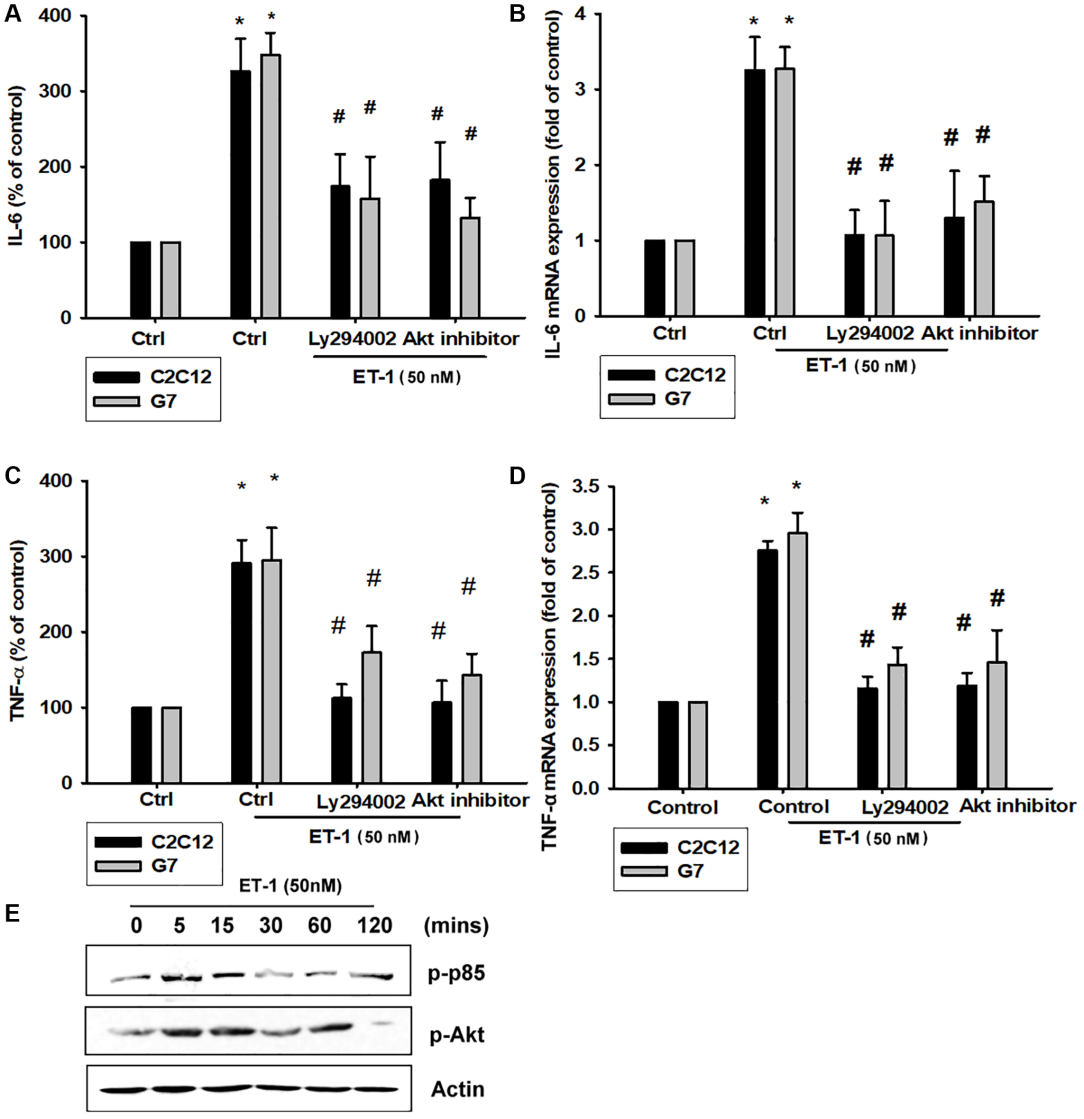
**The PI3K pathway is involved in endothelin-1 or ET-1–induced IL-6 and TNF-α synthesis.** (**A**–**D**) C2C12 and G7 cells were pretreated with a PI3K inhibitor (Ly294002) and an Akt inhibitor then incubated with ET-1 for 24 h. IL-6 and TNF-α levels were examined by RT-qPCR and ELISA (**E**). C2C12 and G7 were incubated with ET-1 for the indicated time intervals, and p85 and Akt phosphorylation were examined using Western blotting. Results are expressed of four independent experiments performed in triplicate. ^*^p < 0.05 compared with control; ^#^*p* < 0.05 compared with the ET-1–treated group.

The PI3K signaling pathway modulates several cellular functions, including inflammation [49]. We explored the role of PI3K in ET-1–enhanced IL-6 and TNF-α expression by pretreating C2C12 and G7 cells with a PI3K inhibitor (Ly294002). When C2C12 and G7 cells were treated with an Akt inhibitor prior to ET-1 administration, we observed marked reductions in ET-1–induced increases in IL-6 and TNF-α expression (Figure 4A–4D). Western blot analysis determined that ET-1 time-dependently promoted p85 and Akt phosphorylation (Figure 4E). These findings suggest that ET-1 facilitates IL-6 and TNF-α production in myocyte cell lines C2C12 and G7 through PI3K and Akt signaling pathways.

### PI3K/Akt/NFκB signaling pathway is involved in ET-1-regulated IL-6 and TNF-α

**Figure 5:**
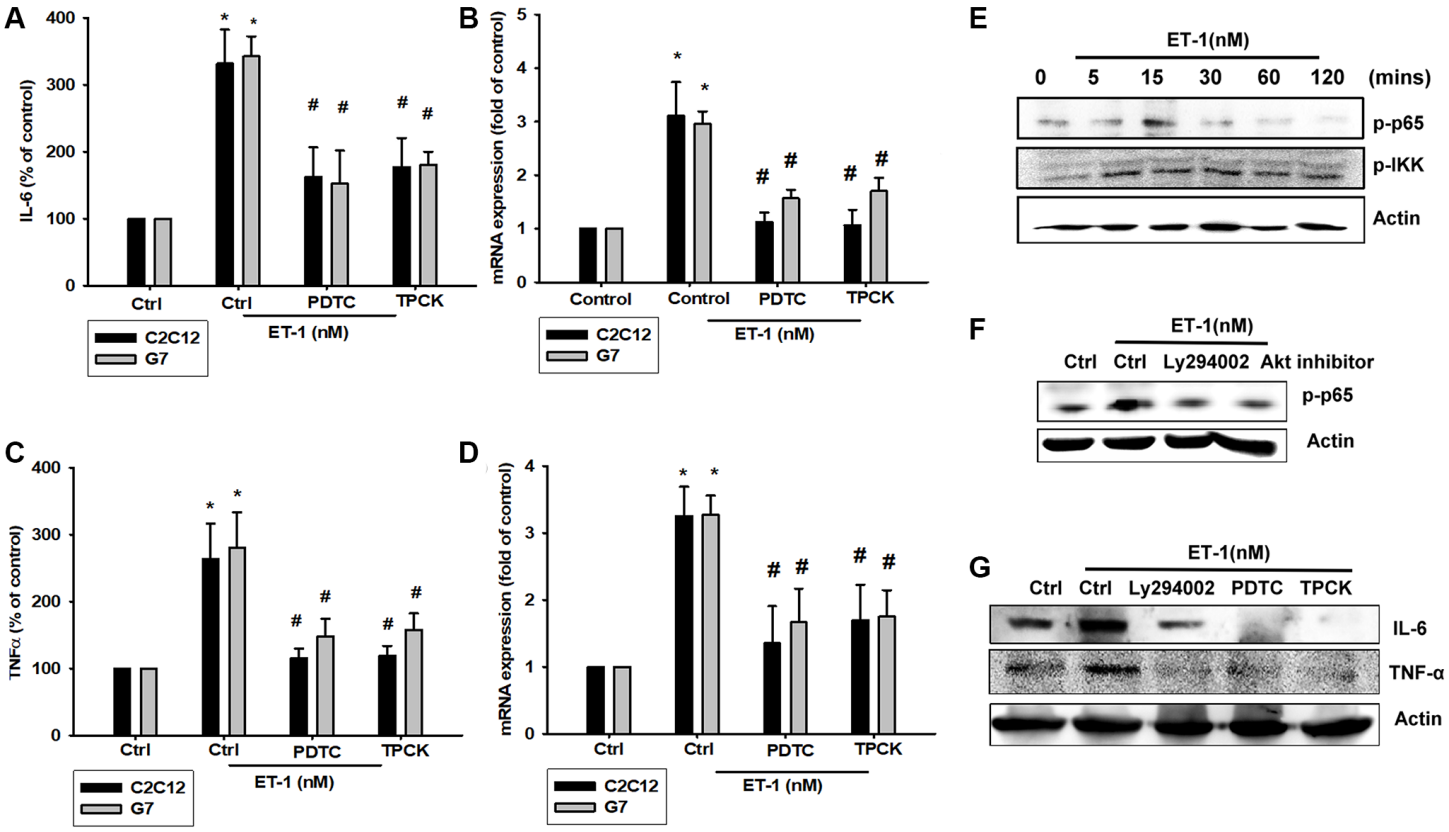
**NFκB is involved in the potentiation of IL-6 and TNF-α production by endothelin-1 (ET-1).** (**A**–**D**) C2C12 and G7 cells were pretreated with NFκB inhibitor PDTC (10 μM) and TPCK (3 μM) then incubated with ET-1 (50 nM) for 24 h. IL-6 and TNF-α levels were examined by RT-qPCR and ELISA. (**E**–**G**) C2C12 were incubated with ET-1 for the indicated time intervals, p65, and IKK phosphorylation were examined using Western blotting. Pretreated with NFκB inhibitor PDTC (10 μM), TPCK (3 μM), and Ly294002 then incubated with ET-1 (50 nM) and p65 phosphorylation and IL-6 and TNF-α were examined using Western blotting. Results are expressed of four independent experiments performed in triplicate. ^*^p < 0.05 compared with control; ^#^*p* < 0.05 compared with the ET-1–treated group.

**Figure 6:**
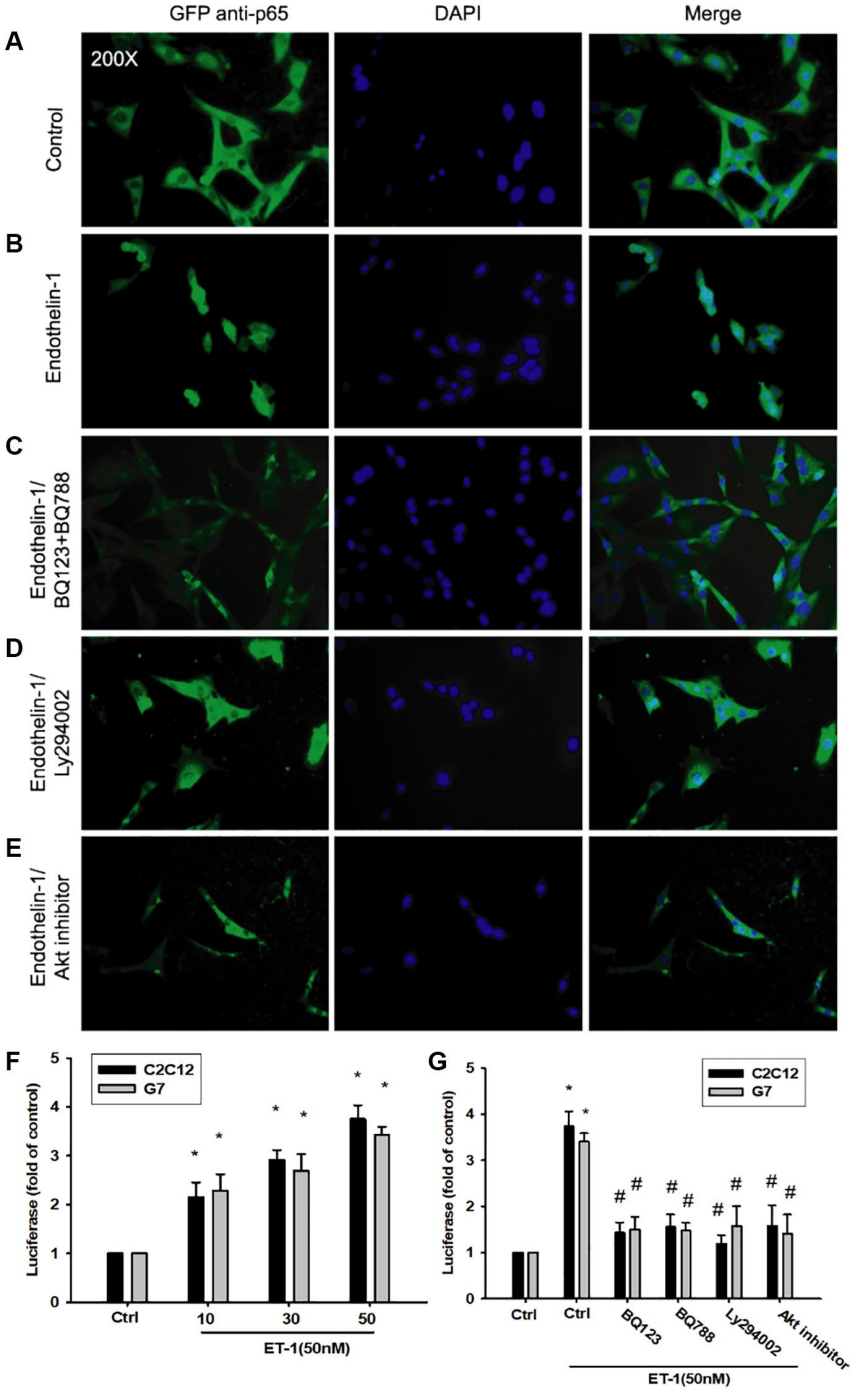
**The PI3K/Akt signaling pathway is involved in endothelin-1 or ET-1–mediated NFκB activity IL-6 and TNF-α expression.** (**A**–**E**) C2C12 and G7 cells were pretreated with BQ123, BQ788, Ly294002, or Akt inhibitor followed, by stimulation with ET-1, and were analyzed using immunofluorescence assay. (**F**) C2C12 and G7cells were incubated for 24 h with various concentrations of ET-1, and *in vitro* NFκB-luciferase activity was measured. (**G**) C2C12 and G7cells were pretreated with BQ123, BQ788, Ly294002, or Akt inhibitor for 30 min stimulated for 24 h with ET-1 (50 nM), and *in vitro* NFκB–luciferase activity was measured. Results are expressed of four independent experiments performed in triplicate. ^*^p < 0.05 compared with control; ^#^*p* < 0.05 compared with the ET-1–treated group.

As mentioned, IL-6 and TNF-α cytokines are released in adipocytes and myocytes through the inflammatory pathway mediated by NFκB activation [50–52]. Treatment of myocyte C2C12 cells with ET-1 also caused. p65 Ser536 phosphorylation has previously been demonstrated to increase NFκB transactivation. Using the same antibody, we determined that treatment of C2C12 cells with ET-1 at various time intervals resulted in IKKα/β phosphorylation (Figure 5E). We further examined the upstream molecules involved in ET-1–induced NFκB activation. Because PI3K/Akt/NFκB was involved in ET-1-induced IL-6 and TNF-α expression, we pretreated the cell lines with the NFκB inhibitor PDTC (10 μM) and IκB protease inhibitor TPCK (3 μM) before treatment with ET-1. They reduced mRNA and protein expression significantly (Figure 5A–5D). Furthermore, pretreatment with upstream pathway inhibitors such as Ly294002 and Akt inhibitor reduced ET-1–activated NFκB pathways (Figure 5F and 5G). These data suggest that NFκB activation is involved in ET-1–induced inflammation in myocyte cells. To directly determine the level of NFκB activation after ET-1 treatment, C2C12 and G7 cells were transiently transfected with κB-luciferase as an indicator of NFκB activation. As depicted in Figure 6F, ET-1 (10–50 nM) treatment of C2C12 and G7 cells for 24 h caused an increase in NFκB-luciferase activity. These results indicate that NFκB activation is essential to ET-1–induced myopathy. To further investigate whether ET-1–induced NFκB activation occurs through the ETRs and PI3K/Akt pathways, C2C12 and G7 cells were pretreated with BQ123, BQ788, Ly294002, and Akt inhibitor, all of which inhibited the ET-1–induced increase in NFκB-luciferase activity (Figure 6G). Stimulation of cells with ET-1 increased p65 translocation into the nucleus, as measured by immunofluorescence staining. The ETRs inhibitor Ly294002 and an Akt inhibitor reduced the ET-1–mediated translocation of p65 (Figure 6A–6E). Taken together, these data suggest that activation of the PI3K/Akt pathway was stimulated by ET-1–induced p65 Ser536 phosphorylation and NFκB activation through ETRs/PI3K/Akt in C2C12 and G7 cells.

### ET-1 induces adipose tissue inflammation and impairment of mitochondria, causing obesity

**Figure 7:**
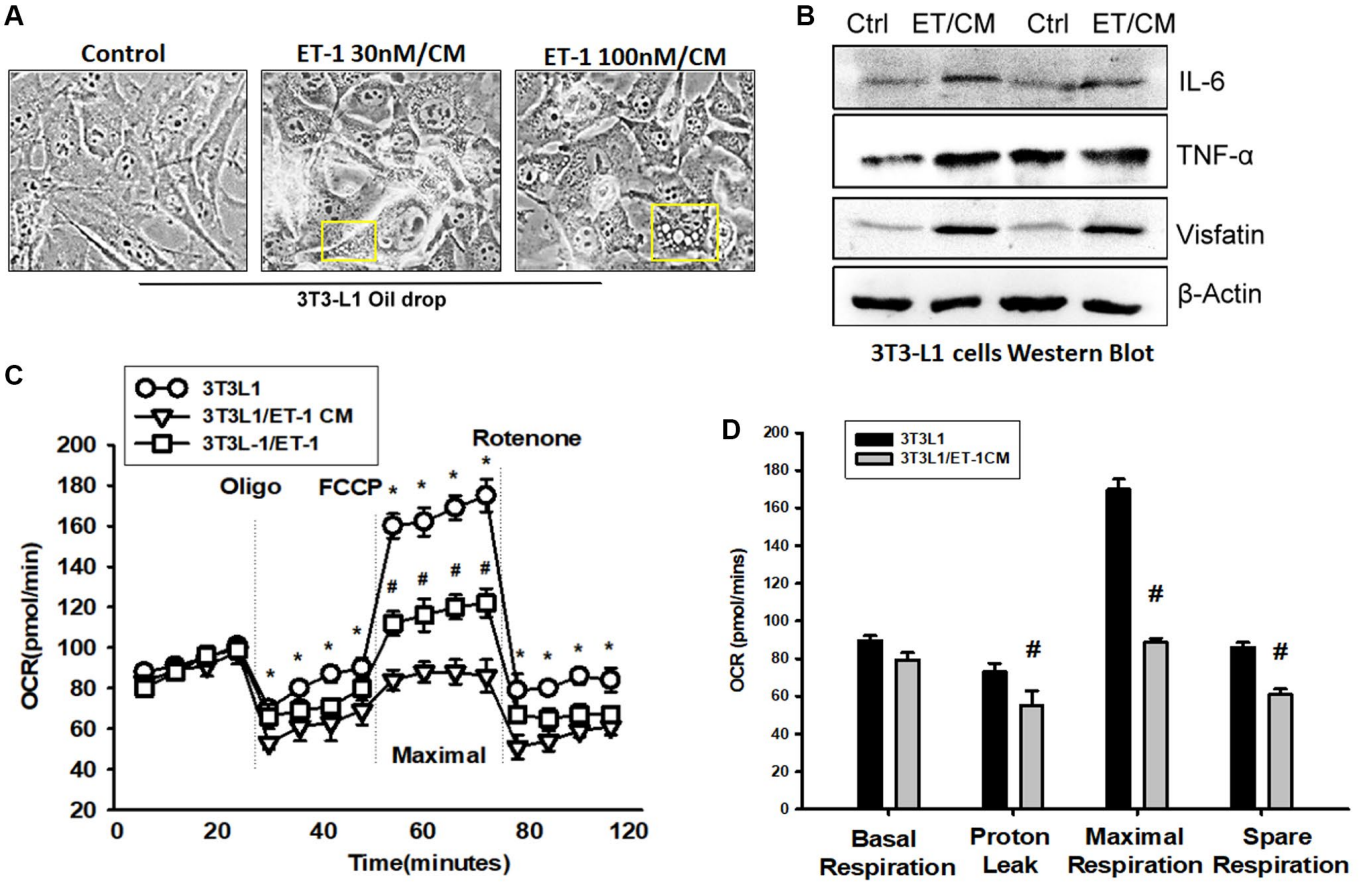
**Endothelin-1 (ET-1) enhances IL-6, TNF-α, and visfatin synthesis and reduces mitochondria oxygen consumption in AT.** (**A**) Bright-field image (100×) of 3T3-L1 preadipocytes cells were incubated with supernatant from C2C12 and observes for 3days with oil-drop. (**B**) Supernatant from C2C12 and G7 treated with ET-1 were incubated with 3T3-L1 cells. *in vitro* IL-6, TNF-α, and visfatin activity were measured through Western blotting. (**C**) 3T3L-1 cell mitochondrial energy measured by Seahorse. Representative OCR plot with the injection of oligomycin, FCCP, and rotenone. (**D**) Quantization data of the different areas of ORC. All the control groups in Figure 7 are from the control CM of C2C12 without treat with ET-1. Results are expressed of four independent experiments performed in triplicate. ^*^p < 0.05 compared with control; ^#^*p* < 0.05 compared with the ET-1–treated group.

Adipose tissue (AT) inflammation resulting in metabolism system impairment is the major reason for obesity. To explore the influence of ET-1 on AT, we directly treated 3T3-L1 adipocyte cells with ET-1. Notably, the expression of cytokines IL-6 and TNF-α and the adipokine visfatin increased in a dose-dependent manner. Furthermore, to modify the condition in human physiology between muscle and AT, we collected CM from ET-1 treated with C2C12 and co-cultured with 3T3-L1 adipocyte cells (Figure 7A and 7B). The data provide strong evidence that ET-1 (or myopathy) mediates cytokine expression, which can promote the release of more inflammatory factors, causing chronic inflammation, and driving fat droplet genesis and storage.

To observe the effect of ET-1 stimulation on the energy metabolism of adipocytes, the promotion of the differentiation of fat cells, or the change in energy metabolism that causes obesity, we analyzed mitochondrial maximum operating capacity (XF Cell Mito Stress Test). To first detect the initial oxygen consumption status, an ATP synthase inhibitor is added to inhibit the mitochondrial production of ATP; the suppressed oxygen consumption indicates how much oxygen is involved in the synthesis of ATP. FCCP is then added. This drug is uncoupled at the appropriate concentration without damaging the electron transport chain, allowing the mitochondria to idle in the limited condition and enabling an evaluation of the maximum oxygen consumption capacity of the mitochondria. Finally, the complex I inhibitor rotenone and complex III inhibitor antimycin A are added to the electron transport chain. Oxygen supply is completely stopped, and the value of its background detection is determined (Figure 7C). Using XF Seahorse Technology, we created a method to directly quantify mitochondrial bioenergetics in 3T3-L1 cells. Our results indicate that decreases in basal oxygen consumption following pretreatment with ET-1 in C2C12/CM are mostly due to proton leakage. In addition, presents lower maximal oxygen consumption than AT (Figure 7D). ET-1 promotes fat metabolism from the original aerobic metabolism to anaerobic metabolism. The results of these experiments suggest that ET-1 promotes the differentiation of AT and energy into storage type, which eventually leads to the obese metabolic pathway. In line with the hypothesis of this study, ET-1 may be responsible for sarcopenia. When adipocytes are stimulated with ET-1, they release related cytokines, such as IL-6, visfatin, and TNF-α. Therefore, we confirmed that ET-1 is a critical mediator of inflammation in myopathy. Normally, it would have a minor and nonsignificant influence; however, a chronic condition could result in amplification in the microenvironment and result in pathology.

### ET-1 enhances NFkB, IL-6, TNF-α expression by inhibiting miR-let-7g-5p synthesis

**Figure 8:**
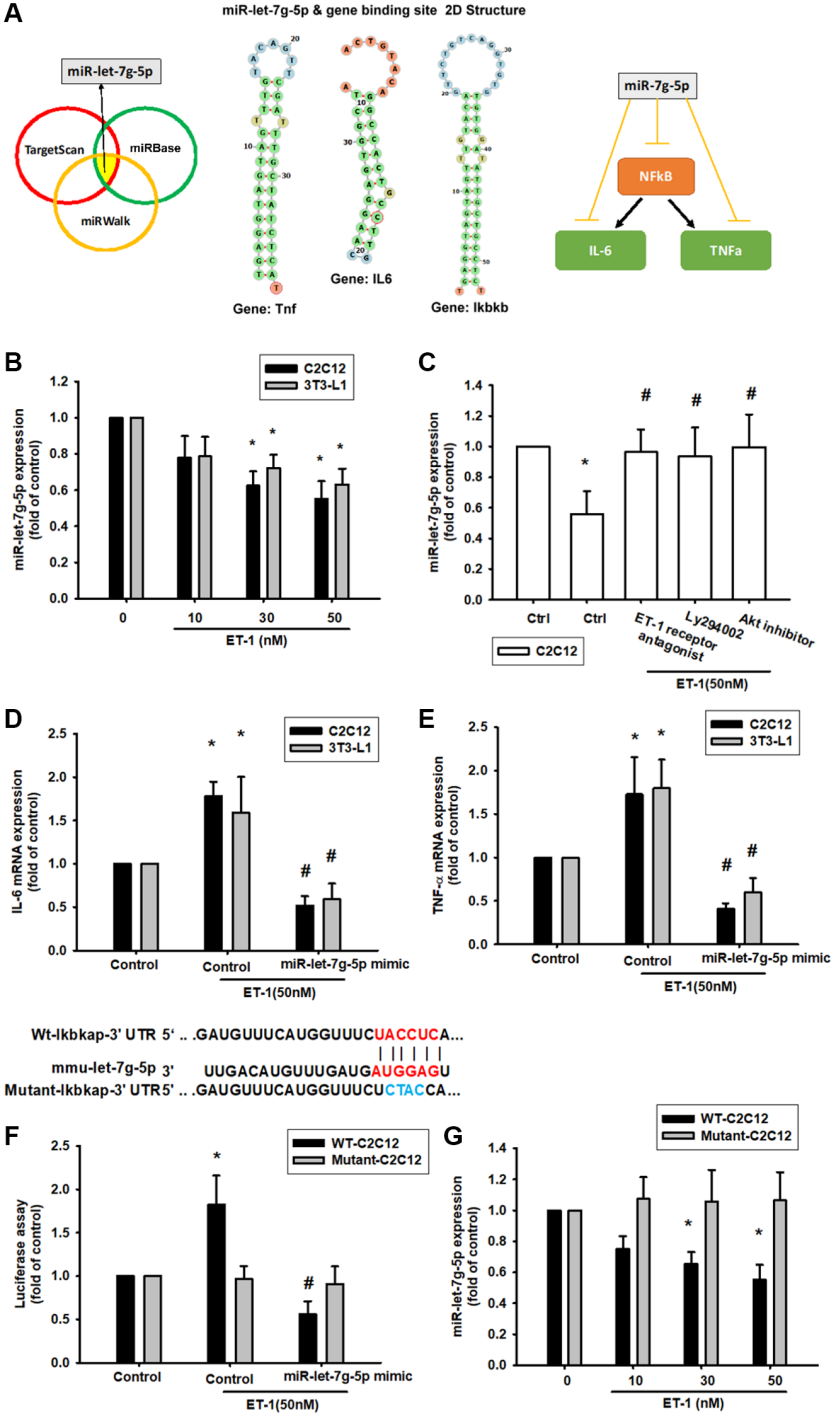
**ET-1 suppresses production of NFkB, TNF-α and IL-6 by increasing miR-let-7g-5p expression.** (**A**) Open-source software (TargetScan, miRDB, and miRWalk) sought to identify miRNAs that could possibly interfere with NFkB, IL-6 and TNF- α transcription. (**B**) Cells were incubated with melatonin (0-50 nM) for 24 h and miR-let-7g-5p expression was examined by qPCR. (**C**) Cells were pretreated with BQ123+BQ788, Ly294002, Akt inhibitor for 30 min, then stimulated with ET-1 for 24 h. miR-let-7g-5p expression was examined by qPCR. (**D**, **E**) Cells were transfected with the miR-let-7g-5p mimic and then treated with ET-1 (50 nM). TNF-α and IL-6 expression was evaluated by qPCR. The wild-type and mutant Ikbkb 3′-UTRs contained the miR-let-7g-5p binding site. (**F**) Cells were transfected with the miR-let-7g-5p mimic and then treated with ET-1 (50 nM). (**G**) Cells were transfected with 3′-UTR plasmids as indicated then stimulated with ET-1 dose concentration. Then, cells were transfected with indicated luciferase plasmids for 24 h then stimulated with ET-1 for 24 h. Relative luciferase activity was measured. Results are expressed as the mean ± SEM. ^*^P < 0.05 compared with controls; ^#^*P* < 0.05 compared with the melatonin-treated group.

The intricate networks of miRNAs in myocyte-induced micro-inflammation are little understood. Our results indicate that ET-1 promotes cell migration by upregulating TWIST expression. We therefore undertook a bioinformatics analysis using open-source database software (TargetScan: http://www.targetscan.org/vert_72/, miRWalk: http://zmf.umm.uni-heidelberg.de/apps/zmf/mirwalk2/, and miRBase: https://www.mirbase.org/) to screen for miRNAs that regulate NFkB, IL-6, TNF-α expression and the 2D statures (Figure 8A). NFkB is the upstream transcription of IL6 and TNF, so we initially searched for miRNAs that would bind at the 3′UTR binding site of NFkB (Ikbkb), and we found that the miRNA of choice was miR-let-7g-5p. Fortunately, after database comparison, we found that this miRNA regulates both IL-6 and TNF and was most substantially inhibited by ET-1 stimulation. To validate these findings, we compared the expression levels of miR-let-7g-5p in C2C12 and 3T3-L1 under graded ET-1 stimulation. We found that ET-1 (1–50 nM) suppressed the extent of miR-let-7g-5p expression in a concentration-dependent manner (Figure 8B). In addition, the ET-1 receptor, PI3K, and Akt inhibitors reversed ET-1-inhibited miR-let-7g-5p expression. miR-let-7g-5p appears to disturb NFkB transcription via binding to the 3′UTR region of NFkB mRNA, and miR-let-7g-5p expression is suppressed by ET-1 receptor/PI3K/Akt phosphorylation induced by upstream ET-1 stimulation (Figure 8C). To define whether ET-1 enhances NFkB, IL-6, TNF-α expression by suppressing miR-let-7g-5p synthesis, we transfected the C2C12 and 3T3-L1 with miR-let-7g-5p, which reversed ET-1-enhanced TNF-α and IL-6 (Figure 8D and 8E). We also used the luciferase reporter vector, including the wild-type 3′UTR of Ikbkb mRNA (wt-Ikbkb-3′UTR) and the vector containing mismatches in the miR-let-7g-5p binding site (mutant-Ikbkb-3′UTR), to define whether miR-let-7g-5p disrupts Ikbkb transcription. We found that miR-let-7g-5p mimic mitigated ET-1-induced luciferase activity in the wt-Ikbkb-3′UTR plasmid, but not in the mt-Ikbkb-3′UTR plasmid (Figure 8F). Also that ET-1 (1–50 nM) suppressed the extent of miR-let-7g-5p expression in a concentration-dependent manner in the wt-Ikbkb-3′UTR plasmid, but not in the mt-Ikbkb-3′UTR plasmid (Figure 8G).

## DISCUSSION

Aging causes chronic inflammation and results in several diseases challenges, such as SO and DM. Andersen et al. (2004) surveyed 36 diabetic patients and determined that the muscle strength of their ankles and knees had degenerated by 17% compared with people without diabetes [53]. Tanaka et al. (2016) uncovered that serum creatinine, HbA1c, and leg muscle mass were negatively correlated [54]. Previous studies have also documented how HbA1c levels are affected in different diseases [55, 56]. The findings of these studies are consistent with the conclusions of this article. We found that in elderly diabetic patients, with longer onset time or higher HbA1c concentration, muscle mass ASM/ht^2^ decreased and blood levels of muscle damage indicators Creatinine and ET-1 increased. The muscle mass and strength of individuals with diabetes are weaker than those in individuals without the disease and are related to the degree of HbA1c and the quality of muscle fibers, both of which are worse than those of ordinary healthy people. The plasma concentration of ET-1 in individuals with diabetes is higher than that in people without it [57]. These pieces of evidence indicate that there is a direct relationship between diabetes and skeletal muscle loss in the elderly population, and this study further hopes to find out the possible key factors affecting the two diseases. Therefore, after constructing an algorithm model through the mesh, it was found that ET-1, Creatinine has a very significant expression in the late stage of the disease in the elderly, which implies that ET-1 may be overexpressed by the influence of diabetes in the elderly, and may therefore dominate the inflammatory response of skeletal muscles.

Since it is difficult for patients with chronic diseases to collect additional samples in addition to serum data, the follow-up research on the mechanism of ET-1 on skeletal muscle inflammation is carried out by *in vitro* methods. The previous study showed that endothelial dysfunction, with increased endothelin-1 (ET-1) synthesis, and sarcopenia, characterized by the loss of muscle mass and strength, are two aging-related conditions. They demonstrated that ET-1-induced senescence and fibrosis in cultured mouse myoblast cells depends on the increase of extracellular FN, that interacting with integrin receptor, induces ILK expression, ROS production, and activation of PI3K-AKT-GSK pathway in old mice and cultured myoblasts [58]. Moreover, obesity (and hyperglycemia) has been also shown to result in increased ET-1 peptide and pre-pro ET-1 gene expression [59]. Thus, it is likely that obesity is both driving the trend for higher ET-1 expression in the obese and obese T2D compared with Lean [60]. These studies suggest that skeletal muscle aging, obesity, and diabetes may all contribute to abnormally elevated ET-1 levels, which is also consistent with our findings and provides a good explanation for the source of excess ET-1. In the past, most of the studies were to study the relationship between ET-1 and ROS. Our study focuses on the release of inflammatory factors induced by ET-1, which causes inflammation in the microenvironment and causes damage to skeletal muscles and fat cells.

Chronic inflammation of AT mediates metabolic syndrome and leads to obesity. ATMs maintain the steady-state of AT. Once cytokines are released by adipocytes, ATMs are induced to cause AT function impairment and increase the risk of diabetes [61]. In addition, obese AT is characterized by excessive production and disturbed capacity to store lipids, which accumulate ectopically in skeletal muscle. Lean or obese AT is affected by two types of phospholipid oxidation that polarize macrophages to antioxidant or pro-inflammatory states, respectively [62]. AT exposure to Th2 cytokines stimulates ATM proliferation. For example, in obesity, IL-6 acts as a Th2 cytokine by stimulating M2 polarization and local ATM proliferation, whereas Th1 cytokines such as TNF-α inhibit local ATM proliferation [63]. Moreover, increased production of visfatin and leptin serum levels may lead to the increased release of Th1 cytokines and result in obesity and its association with diabetes. In these studies, the expression levels of inflammatory factors will directly affect the aging and damage of skeletal muscles and will induce an immune response of adipocytes, promote the abnormal metabolism of adipocytes and lead to obesity.

This study also provides strong evidence that CM of ET-1 incubated with myocytes promotes AT production of IL-1, IL-6, TNFα, and visfatin. These cytokines recruit ATMs and cause the co-occurrence of obesity and diabetes. Brown AT has the unique ability to dramatically increase mitochondrial uncoupled fuel oxidation for thermogenesis in response to adrenergic stimulation. A key parameter in assessing brown adipocyte thermogenic capacity is mitochondrial uncoupling, as determined by respiration [64]. Muscle inflammation is an inevitable process in SO [65, 66]. Our previous study revealed that visfatin is a proinflammatory adipokine that results in obesity and even promotes osteoarthritis [67, 68]. Our current results indicate that ET-1 enhances visfatin levels. These shreds of evidence suggest that ET-1 may be the source of visfatin and a series of inflammatory responses. Furthermore, the mitochondrial energy metabolism underlying maximal oxygen consumption. Prolonged exposure with ET-1 forms a microenvironment for promoting chronic disease. Our study indicated that ET-1 induces IL-6 and TNFα through regulated PI3K/Akt pathways and activity NFkB transcription factor, which provide strong shreds of evidence ET-1 could be a cross-bridge factor in SO and DM.

More and more reports documented that miRNAs related to Sarcopenia or DM patients, especially in the elderly population. Muscle development and homeostasis require a fine gene expression modulation by mechanisms in which microRNAs (miRNAs) play a crucial role. miRNAs modulate key steps of skeletal myogenesis including satellite cells renewal, skeletal muscle plasticity, and regeneration [69]. C2C12 is a satellite myoblast cell line and it is suitable to be the material *in vitro* study in this research. The past study supports a possible connection, expression of several miRNAs, and age-related skeletal muscle declines, such as miR-133b and miR-206 [70]. Interestingly, a recent study shown that miRNA-146a and miRNA155 in saliva provide reliable, non-invasive, diagnostic, and prognostic biomarkers that can be used to monitor periodontal health status among diabetic and non-diabetic patients. Those experiences suggested that miRNA could be a diagnostic and prognostic biomarker. Moreover, this article first provides a miRNA candidate miR-let-7g-5p could be the critical regulator in NFkB, IL-6, and TNFα expression. It is the first article mentioned that miR-let-7g-5p regulated SO and DM in elderly patients. Especially, miR-let-7g-5p comes from the screen open miRNA database website and it can be combined with NFkB related gene Ikbkb, IL-6 gene Il6, TNFα gene tnf the mRNA binding sites. In our research, miR-let-7g-5p specificity activated IL-6 and TNFα expression and only binding with wt-ikbkb mRNA sequences but no effect with mutant sequences by ET-1 stimulation. These data provide strong evidence indicating that PI3K/Akt/miR-let pathways might be the major signaling pathways to regulate inflammatory factors such as IL-6 and TNFα releases.

## CONCLUSIONS

**Figure 9:**
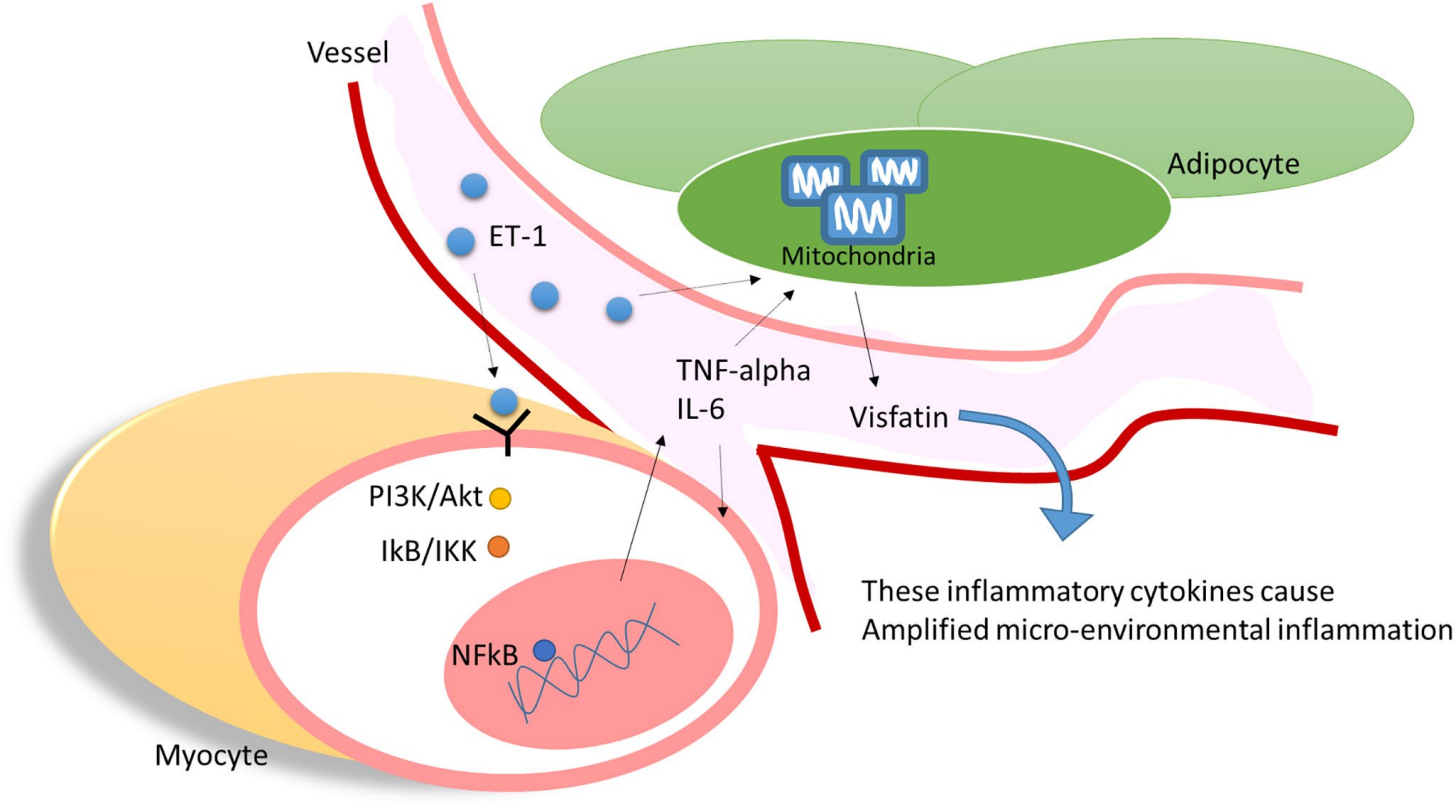
ET-1 played a critical bridging between sarcopenia and DM in elderly patients by triggering the myopathy through myocyte releases inflammation cytokines and affect the adipocyte metabolic meanwhile amplifying microenvironment injury.

Our results indicate that ET-1 may play a critical role in mediating myopathy and adipose inflammation through the release of IL-6, TNF-α, or adipokines visfatin through PI3K/Akt/miR-let-7g-5p pathways in elderly individuals with diabetes (Figure 9). This study provides novel evidence of how ET-1 triggers a systemic inflammatory in hyperglycemic geriatric patients and influences muscle and AT and adds new insights to physiology and pathophysiology. We hope that these findings can promote new thinking and strategies for the treatment of related diseases.
