# Correction for: Endothelin-1-mediated miR-let-7g-5p triggers interlukin-6 and TNF-α to cause myopathy and chronic adipose inflammation in elderly patients with diabetes mellitus

**DOI:** 10.18632/aging.204488

**Published:** 2023-01-15

**Authors:** Chung-Huang Tsai, Pei-Ju Huang, IT Lee, Chien-Min Chen, Min Huan Wu

**Affiliations:** 1 Department of Family Medicine, Chung-Kang Branch, Cheng Ching Hospital, Taichung, Taiwan; 2 Center for General Education Tunghai University, Taiwan; 3 Bachelor of Science in Senior Wellness and Sport Science Tunghai University, Taiwan; 4 Department of Family Medicine, Changhua Christian Hospital, Changhua, Taiwan; 5 Division of Endocrinology and Metabolism, Department of Internal Medicine, Taichung Veterans General Hospital, Taichung, Taiwan; 6 School of Medicine, National Yang Ming Chiao Tung University, Taipei, Taiwan; 7 School of Medicine, Chung Shan Medical University, Taichung, Taiwan; 8 Division of Traditional Chinese Medical, Sinying Hospital, Tainan, Taiwan; 9 Senior Life and Innovation Technology Center Tunghai University, Taiwan; 10 Life Science Research Center Tunghai University, Taiwan

**Keywords:** diabetes, sarcopenia, miRNA, endothelin-1 (ET-1), TNF-α, interleukin-6, hyperglycemia

**This article has been corrected:** The authors found two mistakes in the legend to **Figure 8**:

in the title, “ET-1 suppresses production of NFkB, TNF-α and IL-6 by increasing miR-let-7g-5p expression,” the authors accidentally transposed the words "suppresses" and "increasing." The correct text should be “ET-1 **increases** production of NFkB, TNF-α and IL-6 by **suppressing** miR-let-7g-5p expression”;

in the text of the legend for panel **8B**, the word “melatonin” should be replaced with “ET-1."

The corrected legend to **Figure 8** is presented below.

**Figure 8. ET-1 increases production of NFkB, TNF‐α and IL‐6 by suppressing miR‐let‐7g-5p expression.** (**A**) Open-source software (TargetScan, miRDB, and miRWalk) sought to identify miRNAs that could possibly interfere with NFkB, IL-6 and TNF- α transcription. (**B**) Cells were incubated with ET-1 (0-50 nM) for 24 h and miR‐let‐7g-5p expression was examined by qPCR. (**C**) Cells were pretreated with BQ123+BQ788, Ly294002, Akt inhibitor for 30 min, then stimulated with ET-1 for 24 h. miR‐let‐7g-5p expression was examined by qPCR. (**D**, **E**) Cells were transfected with the miR‐let‐7g-5p mimic and then treated with ET-1 (50 nM). TNF‐α and IL‐6 expression was evaluated by qPCR. The wild-type and mutant Ikbkb 3′-UTRs contained the miR-let‐7g-5p binding site. (**F**) Cells were transfected with the miR‐let‐7g-5p mimic and then treated with ET-1 (50 nM). (**G**) Cells were transfected with 3′-UTR plasmids as indicated then stimulated with ET-1 dose concentration. Then, cells were transfected with indicated luciferase plasmids for 24 h then stimulated with ET-1 for 24 h. Relative luciferase activity was measured. Results are expressed as the mean ± SEM. *P < 0.05 compared with controls; ^#^*P* < 0.05 compared with the melatonin‐treated group.

